# Fully automatic GBM segmentation in the TCGA-GBM dataset: Prognosis and correlation with VASARI features

**DOI:** 10.1038/srep16822

**Published:** 2015-11-18

**Authors:** Emmanuel Rios Velazquez, Raphael Meier, William D. Dunn Jr, Brian Alexander, Roland Wiest, Stefan Bauer, David A. Gutman, Mauricio Reyes, Hugo J.W.L. Aerts

**Affiliations:** 1 Departments of Radiation Oncology and Dana-Farber Cancer Institute, Brigham and Women’s Hospital, Harvard Medical School, Boston, MA, USA; 2 Departments of Radiology, Dana-Farber Cancer Institute, Brigham and Women’s Hospital, Harvard Medical School, Boston, MA, USA; 3 Department of Biostatistics & Computational Biology, Dana-Farber Cancer Institute, Boston, MA, USA; 4 Institute for Surgical Technology and Biomechanics , University of Bern, Switzerland; 5 Department of Biomedical Informatics, Emory University School of Medicine, Atlanta, GA, USA; 6 Support Center for Advanced Neuroimaging (SCAN), Institute for Diagnostic and Interventional Neuroradiology, University Hospital Inselspital and University of Bern, Bern, Switzerland

## Abstract

Reproducible definition and quantification of imaging biomarkers is essential. We evaluated a fully automatic MR-based segmentation method by comparing it to manually defined sub-volumes by experienced radiologists in the TCGA-GBM dataset, in terms of sub-volume prognosis and association with VASARI features. MRI sets of 109 GBM patients were downloaded from the Cancer Imaging archive. GBM sub-compartments were defined manually and automatically using the Brain Tumor Image Analysis (BraTumIA). Spearman’s correlation was used to evaluate the agreement with VASARI features. Prognostic significance was assessed using the C-index. Auto-segmented sub-volumes showed moderate to high agreement with manually delineated volumes (range (r): 0.4 – 0.86). Also, the auto and manual volumes showed similar correlation with VASARI features (auto r = 0.35, 0.43 and 0.36; manual r = 0.17, 0.67, 0.41, for contrast-enhancing, necrosis and edema, respectively). The auto-segmented contrast-enhancing volume and post-contrast abnormal volume showed the highest AUC (0.66, CI: 0.55–0.77 and 0.65, CI: 0.54–0.76), comparable to manually defined volumes (0.64, CI: 0.53–0.75 and 0.63, CI: 0.52–0.74, respectively). BraTumIA and manual tumor sub-compartments showed comparable performance in terms of prognosis and correlation with VASARI features. This method can enable more reproducible definition and quantification of imaging based biomarkers and has potential in high-throughput medical imaging research.

Glioblastoma multiforme (GBM) is the most frequent malignant brain tumor in adults and it is among most lethal primary tumors, characterized by an unfavorable prognosis with a 5-year survival rate of 5%^1^. According to the Cancer Genome Atlas Research Network, GBM tumors should be considered as different entities with distinct molecular characteristics, each with different response to treatment^2^.

Studies have shown that phenotypic features, captured non-invasively by structural magnetic resonance (MR) imaging, are able to stratify GBM patients into distinct prognosis groups^3^,^4^. For instance, quantitative measurements of the relative sub-division of tumors into the non-enhanced, contrast-enhanced and necrotic portions have been associated with response to treatment and prognosis^5^,^6^,^7^.

Also radiogenomic analyses have linked specific MR phenotypes to gene expression profiles in GBM, where molecular characteristics of tumors can be predicted by extraction of characteristic MR features of the necrotic core and the contrast-enhanced tumor rim^8^,^9^,^10^,^38^. However, these features have been frequently assessed qualitatively, i.e., by scoring “presence or absence of enhancement” or by manual delineation of the tumor volumes and sub-compartments, both of which are influenced by inter-rater variability. Particularly in MR images, the inter-observer agreement of GBM boundaries is troublesome^11^.

To elucidate meaningful associations between imaging features and survival or genomic alterations of GBM, accurate and reproducible measurement of MR features is critical^12^,^13^. Automatic segmentation methods reduce inter-observer variability in tumor definition and allow for more reproducible quantification of imaging based tumor metrics^14^,^15^,^16^. Efforts such as the VASARI Research Project have enabled a controlled vocabulary to describe the morphology of GBM on MR images^17^.

Recently, a dedicated fully automated segmentation tool for brain tumors (BraTumIA), has shown to provide statistically equivalent tumor volumes to those manually defined by medical experts, in terms of volume and spatial overlap^18^. However, the potential to define prognostic tumor sub-volumes in MR images needs to be evaluated. Zhang *et al.* proposed an automated segmentation algorithm to define proportions of MR-GBM sub-volumes, which Mazurowski *et al.* associated with survival on 68 TCGA patients, however without comparison with expert defined sub-volumes or external validation of the segmentation algorithm^19^,^20^.

We hypothesize that automatic MR based sub-volume tumor segmentation of GBM will result in comparable prognostic power and association with VASARI data, compared to manually defined sub-volumes.

The purpose of this study is to evaluate the prognostic value of GBM tissue compartments segmented fully automatically using BraTumIA as compared to those manually delineated by expert physicians in the TCGA-GBM dataset^21^. BraTumIA allows fully automatic segmentation of the four relevant tumor sub-compartments: enhancing region, non-enhancing region, necrotic core and edema, and eliminates user interaction and therefore intra – and inter – observer variability.

Furthermore, we compared the resulting automatically segmented tumor sub-volumes with the VASARI^17^ features for their association with survival. Because BraTumIA is publicly available^22^ and easily accessible by download, its application in GBM could have a large impact in high-throughput data mining evaluations of imaging biomarkers in GBM such as Radiomics and Radiogenomics^23^,^24^,^25^.

## Materials and Methods

### Patient selection

MRI sets were downloaded from the Cancer Imaging archive (TCIA) for 109 patients. This is a subset of the patients that were included in the Cancer Genome Atlas project^26^. MR imaging sets included T1w, post-contrast T1w, T2w and fluid-attenuated inversion recovery (FLAIR). All MR imaging sets were reviewed to confirm pre-surgery status. All images have been previously de-identified and are publicly available, therefore no Institutional Review Board approval was required.

### Automated segmentation

**Figure 1:**
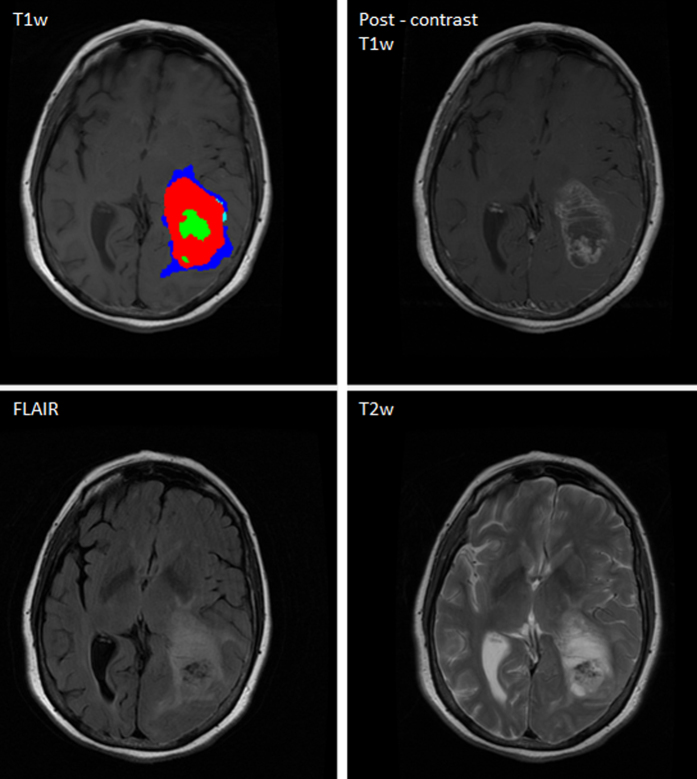
Graphical user interface of the BraTumIA software. After loading the required MR set (T1w, post contrast T1w, T2w and FLAIR), skull stripping, multimodal registration and classification are performed without user interaction. An overlay of the segmentation results is shown for a representative patient (Clockwise: T1w, post-contrast T1w, T2w and FLAIR).

The Brain Tumor Image Analysis (BraTumIA) software was employed for automatic segmentation of MR sets. The software requires the user to load the four respective MRI modalities (T1, post-contrast T1, T2, and FLAIR). First, the images are preprocessed, which encompasses skull stripping followed by multimodal rigid image registration (where the post-contrast T1-weighted image serves as template). Second, segmentation is performed via voxel-wise tissue classification. Every voxel is classified into either unaffected tissue (CSF, GM, WM) or tumor tissue (necrosis, edema, contrast enhancing and non-enhancing tumor). This process can be subdivided into three subsequent stages: a feature extraction yielding a voxel-wise feature vector, a supervised classification step and a spatial regularization. The feature vector is composed of appearance-sensitive (multimodal intensities and intensity differences, first-order and gradient textures) and context-sensitive features (atlas-normalized coordinates, multi-scale symmetry features and ray features). The classifier is a decision forest that outputs for every voxel and tissue type a corresponding probability. The predicted tissue class label of a voxel is chosen as the one with the highest probability. The regularization serves as a refinement of previously generated label maps and is conducted in a hierarchical manner^27^. It is formulated as an energy minimization problem of a conditional random field, where the approximate global optimum corresponds to the final segmentation thus guaranteeing repeatability of results^27^,^28^. After segmentation, label maps can be overlaid on MR images. In Fig. 1, a representative tumor segmentation of a TCIA case is shown.

Prior to the application to new patient data, the segmentation method was trained on 56 high-grade glioma patient image sets not included in the present study. These image sets encompassed the training data used in the study of Porz *et al.*^18^, and an additional set of 20 patient image sets of the Inselspital, University Hospital Bern. The training data was manually segmented by expert radiologists according to a pre-defined protocol^29^. This stand - alone trained BraTumIA model was applied directly to the 109 MRI sets included in this study without user interaction, model re-training or needed previous expertise.

The segmentation method of BraTumIA was ranked among the top performing methods at the MICCAI Brain Tumor Segmentation Challenges (BRATS) 2012 and 2013^30^. Moreover, it belongs to the fastest automatic methods for brain tumor segmentation to date with an average computation time of less than 5 minutes^31^.

### Manual delineations

The software platform Velocity AI (Atlanta, GA) was used to manually delineate, slice-by-slice, a region containing the abnormal signal detected in the post-contrast T1w images, which included the contrast enhancing and necrotic regions for 109 patients.

Similar regions of interest were manually drawn for FLAIR images, encompassing the tumor’s total abnormal signal detected by FLAIR, including all the bright areas indicative for edema as well as abnormal regions. The FLAIR region of interest was restricted to the ipsilateral side of the tumor and did not include signals that may be originated due to ventricular spread, aiming to estimate the overall tumor involvement.

Contrast enhancement and necrosis volumes were determined from the contours drawn on the post-contrast T1w images on an assisted mode using FAST^32^. This algorithm segments the initial post-contrast T1w contours into binary images, using a k-means clustering algorithm, resulting in a binary tissue classification of the post-contrast T1w masks into the contrast enhancing volume and necrosis volume, based on relative pixel intensity. All resulting contrast enhancing and necrotic volumes are manually edited on a slice-by-slice basis.

**Figure 2:**
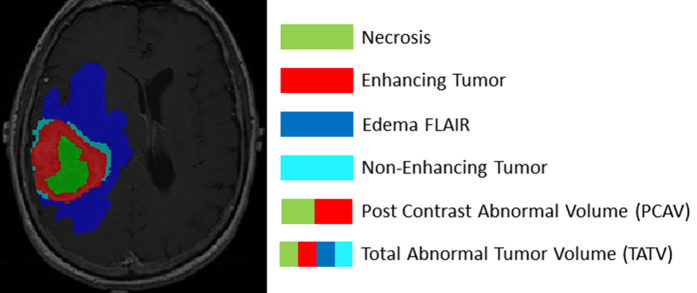
Tumor sub-compartments as defined by the BraTumIA software. A post contrast abnormal volume (PCAV) was defined as the sum of the necrotic and contrast enhancing volumes. The total abnormal volume (TATV) was defined as the sum of the four sub-compartments: necrosis, enhancing region, non-enhancing region and edema. Note that the non-enhancing tumor region was not manually contoured and therefore the TATV manual was defined as the sum of necrosis, enhancing and edema regions.

A post contrast abnormal volume (PCAV) was defined as the sum of the necrotic and contrast enhancing volumes. The total abnormal volume (TATV) was defined as the entire abnormal signal in the FLAIR images. The edema region (FLAIR envelope) was defined as the difference of the PCAV from the TATV (Fig. 2).

### Statistical analysis

Volumetric agreement between the automatically segmented volumes and manual contours was assessed using Spearman rank correlation for all volumes. Because the non-enhancing tumor region was only defined by the automatic segmentation method, we re-defined the automatic edema volume as the sum of auto edema and auto non-enhancing regions, for comparison with the manual edema volume.

We also compared proportion features derived from the automatically segmented volumes against semi-quantitative measurements scored using the VASARI visual feature guide^3^. Four relevant VASARI features were included: proportion of enhancing tumor, proportion of non-enhancing tumor, proportion of necrosis and proportion of edema. To allow this comparison, the percent of enhancing tumor (enhancing volume/TATV), percent of non-enhancing tumor (non-enhancing volume/TATV), percent of necrosis (necrosis volume/TATV) and percent of edema (edema volume/TATV) were defined for the automatically segmented volumes. Because the VASARI features are scored as ordinal variables (e.g. enhancing volume is <5% of the entire abnormality, all categories are: <5%, 6–33%, 34–67% and 68–95%)^17^, we used the Spearman rank correlation to compare the agreement between the VASARI features and the proportion features generated from the automatically segmented tumor sub-compartments.

Next, the prognostic performance of the automatic or manually segmented volumes on pre-operative MRI was assessed using the concordance index (CI), which is the probability that among two randomly drawn samples, the sample with the higher risk value has also the higher chance of experiencing an event (e.g., death) and takes time to event and censoring into account.

As endpoints we used overall survival and 1 year survival calculated from the start of treatment, a clinically important endpoint for GBM patients. For the 1 year survival endpoint, data were excluded if patients were alive and their follow-up time was less than one year (n = 10). 1 year survival predictive performance was evaluated using the area under the curve (AUC) of the receiver operator characteristic (ROC). Additionally, we reported the lower and upper bounds of the confidence interval for all CI/AUC values.

To assess if a CI/AUC is significantly different than random we used the “noether” method as implemented in the R survcomp package^33^. To assess if a CI/AUC was higher than another we used a one-sided t-test (for the comparison cindex1 >cindex2) for dependent samples, as implemented on the cindex.comp function of the R survcomp package.

To evaluate if the different sub-volumes had complementary information in a multivariate setting, we used a minimum redundancy maximum relevance (mRMR) algorithm implemented in the mRMR R package^34^ on all sub-volumes with respect to one year survival to select a non-redundant highly informative set of complementary features.

The mRMR algorithm returns a univariate rank of features; we trained logistic regression models adding features one by one starting by the highest ranked feature. We cross-validated the performances of each model with resamples and retained the model with the highest mean AUC.

Statistical analyses were performed in R (R Foundation for Statistical Computing, Vienna, Austria).

## Results

### Volumetric agreement between automatic and manual volumes

**Figure 3:**
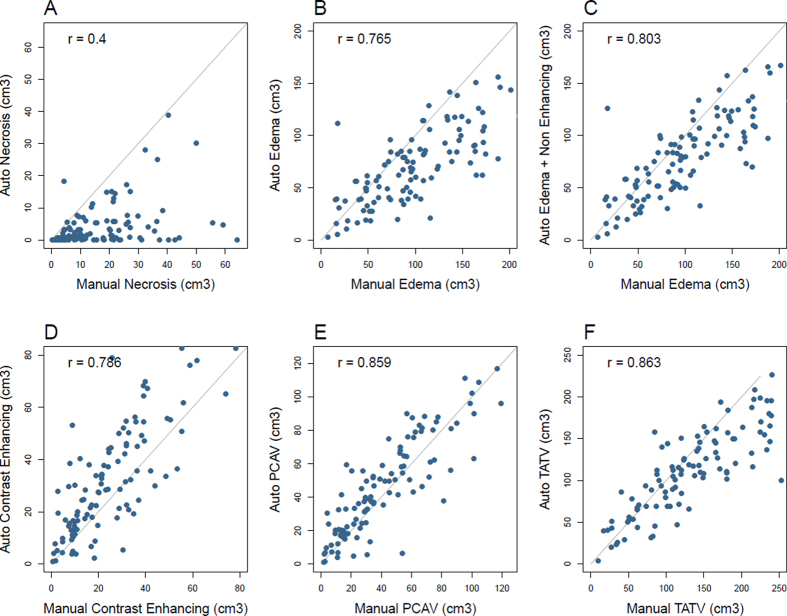
Scatter plots showing the correlation between different tumor regions as defined fully automatically or manually drawn. Necrosis showed the weakest correlation ((**A**), r = 0.4). Stronger correlations were found for the FLAIR envelope and contrast enhancing regions, ((**B**), r = .765) and ((**D**), r = 0.786), respectively. PCAV (necrosis + enhancing regions) and the TATV (sum of all sub-compartments), r = 0.86 and r = 0.86, showed strong correlation as well between automatic and manual segmentation (**E,F**).

We investigated the correlation between the automatic and manual techniques to define the tumor sub-compartments: necrosis volume, contrast enhancing volume, edema volume (FLAIR), and the composed post-contrast abnormal volume and the total abnormal tumor volume. Scatter plots depicting these correlations are shown in Fig. 3.

The Spearman’s rank correlation between both segmentation techniques for defining the necrosis volume was *r* = 0.4. High Spearman’s correlations were observed for the contrast enhancing and FLAIR envelope regions, *r* = 0.79 and *r* = .77, respectively. Because the non-enhancing tumor was not manually defined, we compared the manual edema region with the sum of the auto-segmented non-enhancing and edema regions. This resulted in an increased correlation of *r* = 0.80, and suggests that in terms of volumetric association, the sum of auto edema and non-enhancing regions reflects more closely the manually defined edema sub-volume.

Strong correlations were observed for both tumor-definition methods for the PCAV (necrosis + enhancing regions) and the TATV (sum of all sub-compartments), *r* = 0.86 and *r* = 0.86, respectively.

**Figure 4:**
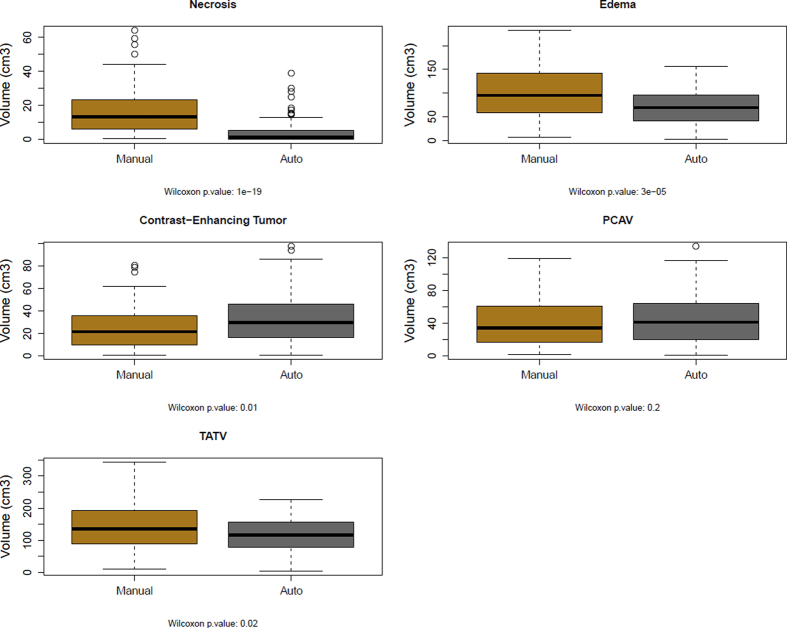
Raw volumes in mm^3^ as determined by manual delineation and by the fully auto-segmentation. Statistical differences were assessed using the Wilcoxon test.

Figure 4 shows a comparison of the raw volumes for all tumor-sub compartments and edema for both tumor definition methods. Statistically significant differences were observed for necrosis, the contrast-enhancing tumor, edema and TATV, between both segmentation methods. Overall, the auto segmentation method defined significantly smaller necrotic and edema volumes and significantly larger contrast enhancing volumes, as compared to the manual ones. No statistical differences were observed for the post-contrast abnormal volume.

### Comparison with VASARI features

The VASARI features were available for 80 patients. To evaluate the correlation between the auto-segmented volumes and the VASARI features, four relevant percent statistics were calculated: proportion of enhancing tumor, proportion of non-enhancing tumor, proportion of necrosis and proportion of edema.

We used the TATV as denominator to determine the sub-volume relative proportions.

The Spearman correlations for the proportion of contrast-enhancing, necrosis and edema with their respective VASARI features were: r = 0.35, 0.43 and 0.36 (p < 0.001). The non–enhancing proportion of the tumor did not show a correlation with its corresponding VASARI feature (r = −0.10; p = NS).

Additionally we evaluated if the manual volumes are associated with the VASARI features. The Spearman’s rank correlations with the VASARI features were 0.17 (p = NS), 0.67 (p < 0.001) and 0.41 (p < 0.001), for the percentage of contrast enhancing, percentage of necrosis and percentage of edema, respectively.

### Fully automatic vs manual: prognostic analysis of tumor sub-compartments

The median follow up of all patients was 10.9 months (range: 0.2–56.2 months). At the time of last follow-up, 81.5% of the patients had deceased and 18.5% were alive. One year survival rate was 44.4%. One case was excluded as survival and follow-up times were not available.

The prognostic performance of the automatic and manual volumes was determined using the C-index. For the 1 year survival endpoint, we used the area under the curve (AUC) of the receiving operating characteristic (ROC), which is identical to the C-index but can be used for binary classifications.

**Figure 5:**
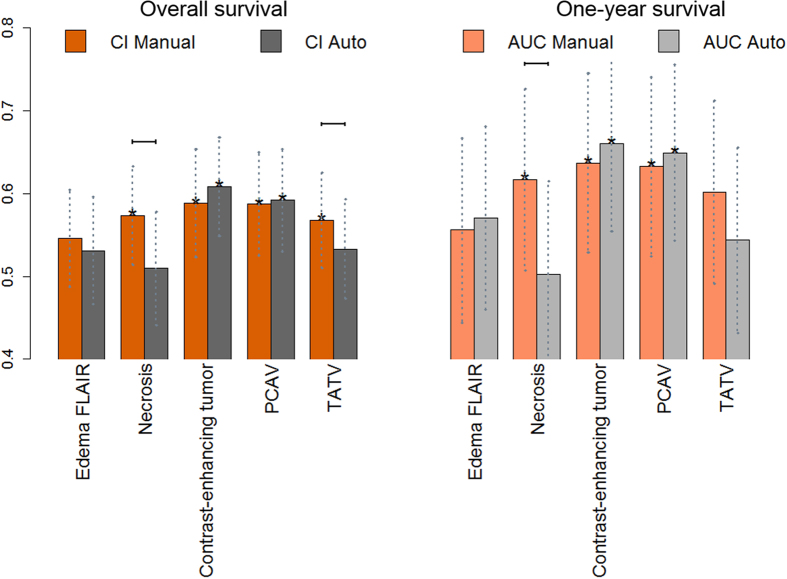
Prognostic performance (C-index, n = 108/AUC, n = 98) for all tumor sub-compartments and composed volumes for the fully automatic segmentation algorithm and manual delineations. The concordance index (CI) is a generalization of the AUC, which takes into account the time to event and censoring, and provides the probability that among two randomly drawn samples, the sample with the higher risk value has also the higher chance of experiencing an event (e.g., death). The symbol * denotes that a CI/AUC is significantly different than random (CI/AUC = 0.5) and was determined using the “noether” method as implemented in the R survcomp package^33^. Vertical dotted lines indicate confidence intervals for the CI/AUC values. Horizontal solid lines indicate that a performance metric (CI or AUC) is significantly higher for the indicated segmentation method as compared to its counterpart (p < 0.05, p-value from the Student t test for the comparison cindex1 > cindex2 as implemented in the R suvcomp package).

Figure 5 summarizes the comparison between the manual and auto-segmented volumes in terms of outcome prediction. Overall, estimation of 1 year survival showed higher AUC values than prediction of overall survival.

Contrast enhancing volume and PCAV were significantly prognostic for both segmentation methods. Necrosis was significantly prognostic only for the manual contours. For necrosis, both prognostic performance measures were significantly higher for the manual volume as compared to the automatic volume.

Edema (FLAIR) did not show significant prognostic value for any of the segmentation techniques. Interestingly, the TATV which encompasses all tumor sub-compartments and the edema did not show significant prognostic power, except for the manual TATV C-Index. This underlines the importance of performing a meaningful sub-division of the entire GBM lesion into distinct sub-compartments with distinct prognostic implications.

The highest AUC values were observed for the contrast enhancing region (AUC = 0.66, *p* < 0.001) and PCVA (AUC = 0.65, *p* = 0.01) as defined by the auto-segmentation algorithm. However, these AUCs were not significantly higher than those estimated for the manual contrast-enhancing and PCVA volumes. The non-enhancing region, only segmented by the automatic technique, did not show significant CI or AUC values, 0.54 (*p* = 0.2) and 0.60 (*p* = 0.08), respectively.

Differences in C-index or AUC between the two segmentation methods were not significant for the evaluated volumes (see overlapping confidence intervals in Fig. 5), with the exception of the necrotic sub-volume and the TATV, meaning that volumes showed a comparable prognostic value for the automatic and manual segmentations.

The mRMR selection resulted in the auto contrast enhancing sub-volume as the only feature selected in the multivariate logistic-regression setting with a mean AUC of 0.67. Adding other sub-volumes (either manually or automatically selected) did not improve the logistic regression model accuracy.

## Discussion

Medical imaging is moving towards a quantitative computational science, where automated methods for tumor definition and characterization are increasingly being developed and applied^23^. A recently developed fully automatic segmentation algorithm (BraTumIA) has shown to provide GBM tumor sub-compartments statistically equivalent, in terms of extent and spatial location, to those manually defined by medical experts in MR images^18^. This study focuses on evaluating as endpoint the prognostic implications of the segmented GBM sub-volumes. Therefore we evaluated whether GBM sub-compartments defined by the BraTumIA automatic method are comparable in terms of prognosis to the sub-volumes defined manually by experienced radiologists in the TCGA-GBM dataset.

Extensive literature has reported on the inter-observer variability with respect to tumor definition in GBM^14^. Previous studies emphasized the importance of minimizing user interaction in order to reduce inter-observer variability^12^,^16^. Because BraTumIA is a fully automated segmentation tool, it eliminates user interaction and thus intra – and inter – observer variability. Imaging based metrics, i.e. the contrast enhancing portion of the tumor in pre-operative MR images, have been associated with patient prognosis and response to therapy^3^,^5^,^6^,^7^. A crucial step for the development of imaging-based markers is to enable their reproducible definition and quantification, as well as to evaluate their importance for patient prognosis and response to therapy. Therefore, a fully automatic segmentation tool for GBM, becomes an attractive method to identify MR based prognostic markers. Zhang *et al.* proposed an automatic segmentation algorithm developed and evaluated on 73 TCGA patients, which was compared with manual references in terms of volumetric correlation^20^. Mazurowski *et al.* applied this method to 68 TCGA patients and evaluated automatically defined GBM features for prediction of survival. This study did not compare the automatically defined volumes with those defined by medical experts in terms of prognosis^19^. Both studies lacked validation as their methods were developed and evaluated using the same set of patients, which limits their generalizability and makes them prone to overfitting and inflated type-I errors^35^.

This is to our knowledge, the first study comparing the prognostic performance of automatically segmented GBM sub-compartments with their manually defined equivalents. We showed that the contrast enhancing volume and the post contrast abnormal volume (necrosis + contrast enhancing) had the highest ability to predict overall and one year survival. However these predictive values were not significantly higher than those obtained with the manual sub-volumes. Interestingly, the total abnormal tumor volume, encompassing necrotic, enhancing and non-enhancing tumor portions plus edema did not show a significant prognostic power, except for the manually defined TATV CI. This drop in prognostic performance may be driven by the edema, which was not prognostic for both methods. This emphasizes the importance of distinguishing the GBM tumor sub-compartments in terms of prognosis.

Necrosis was prognostic for the manually defined volume only. We observed as well that the necrotic region was significantly smaller for the auto-segmentation algorithm, as compared to the manual volumes. However the manual definition of necrosis was done by a binary classification of the initial manually delineated region encompassing the necrotic core and the contrast-enhancing tumor region. This binary classification was performed by applying an intensity threshold that varies across patients and inevitably introduces variability to the definition of the necrotic and contrast-enhanced sub-volumes. This may also explain why the manual necrotic volumes are significantly larger than the automatic ones, while conversely the contrast-enhanced volumes are significantly smaller for the manual method.

Overall, the sub-volumes segmented automatically appeared to be comparable in terms of prognosis to the manually delineated sub-volumes. The mRMR-based multivariate feature selection indicated that the auto contrast-enhancing sub-volume had the highest predictive accuracy and that adding other volumes (either manually or automatically segmented) did not improve the prediction accuracy.

We compared the auto-segmented volumes with relevant VASARI features. The results showed that the non-enhancing relative proportion of the tumor showed no correlation with the VASARI non-enhancing proportion. This perhaps due to the difficulty, even for expert raters, to define the non-enhancing tumor volume. The correlations for the percent of necrosis, contrast-enhancing and edema with their corresponding VASARI features ranged from weak to moderate, however statistically significant in all cases. This correlation was not significant for the manual contrast-enhancing portion, while it was stronger for the manual necrotic portion and its corresponding VASARI feature.

The dataset evaluated in this study is a subset of a larger cohort of patients included in the TCGA consortium^26^, that involves a number of institutions with different image acquisition protocols and different scanners, representing the variability frequently observed in the routine clinical practice, and therefore is remarkable that the fully automated segmentation, which does not require user intervention nor previous expertise, renders significantly prognostic volumes, as good as the manually delineated by medical experts. Furthermore, the data used for training BraTumIA was manually segmented by different expert raters and according to a different segmentation protocol than the TCGA data used for evaluation. This corresponds to a realistic clinical scenario and highlights BraTumIA’s capability to generalize.

A limitation of this algorithm is that it has been developed for pre-operative MR sets only. Future developments should involve its extension to longitudinally acquired imaging sets that would enable volumetric measurements of tumor response to treatment, i.e. evaluation of radiological response rates and the quantification of the extent of tumor resection^36^ which has been associated with patient favorable prognosis^37^. Furthermore, its application is limited to patient sets that include all four required MR modalities: T1w, post-contrast T1w, T2w and FLAIR.

The end goal of auto-segmentation algorithms is to enable reproducible definition and quantification of imaging based markers that have prognostic implications. Thus further evaluation of this algorithm will involve larger patient cohorts, evaluation of its influence in defining intra-tumoral imaging metrics as well as longitudinal analyses of tumor response to treatment.

## Additional Information

**How to cite this article**: Velazquez, E. R. *et al.* Fully automatic GBM segmentation in the TCGA-GBM dataset: Prognosis and correlation with VASARI features. *Sci. Rep.* **5**, 16822; doi: 10.1038/srep16822 (2015).
